# Guarding the gatekeepers: a comprehensive approach to control nosocomial measles

**DOI:** 10.1007/s15010-024-02186-0

**Published:** 2024-02-14

**Authors:** Andrew Limavady, I.-Ting Tu, Helen Bedford

**Affiliations:** 1 Great Ormond Street Institute of Child Health, University College London, London, UK; 2 Population, Policy and Practice Department, UCL Great Ormond Street Institute of Child Health, London, UK

**Keywords:** Measles, Healthcare workers, Nosocomial transmission, Control, Preventive strategies

## Abstract

**Purpose:**

Despite substantial vaccination progress, persistent measles outbreaks challenge global elimination efforts, particularly within healthcare settings. In this paper, we critically review the factors contributing to measles outbreak and effective control measures for nosocomial transmission of measles.

**Methods:**

We systematically searched electronic databases for articles up to 17th May, 2023. This was performed by two independent reviewers, with any disagreements resolved by a third reviewer. We also searched governmental and international health agencies for relevant studies.

**Results:**

Forty relevant articles were systematically reviewed, revealing key factors fuelling measles outbreak in healthcare settings, including high transmissibility capability; high intensity exposure; delayed care; failure to use protective equipment and implement control measures; vaccine failure; unclear immunisation history and lack of registries; and lacking recommendation on healthcare workers’ (HCWs) measles vaccination. To combat these challenges, successful control strategies were identified which include early notification of outbreak and contact tracing; triaging all cases and setting up dedicated isolation unit; strengthening protective equipment use and physical measures; improving case detection; determining immunity status of HCWs; establishing policy for measles vaccination for HCWs; management of exposed personnel; and developing a pre-incident response plan.

**Conclusion:**

A coordinated and comprehensive approach is essential to promptly identify and manage measles cases within healthcare settings, necessitating multifactorial strategies tailored to individual settings. These findings provide a valuable foundation for refining strategies to achieve and maintain measles elimination status in healthcare environments.

## Introduction

Measles is a systemic viral infection caused by paramyxovirus that spreads by direct contact with respiratory secretions or droplets. Despite its high transmissibility (basic reproduction number of 12–18) [1], implementation of measles vaccine programme since the late 1960s with inclusion of the two-dose vaccine regime in almost all countries has resulted in a significant reduction in measles incidence [2, 3]. Even one dose of measles, mumps, and rubella (MMR) vaccine is over 80% effective in preventing the spread [4]. Ma and colleagues [5] reviewed the implementation of the Chinese Action Plan, which included achieving 95% vaccination coverage, intensive laboratory surveillance, and confirmation of confirmed cases, all resulting in a substantial decrease in measles incidence from 99.4 in 2008 to 4.6 per million population by 2012. A coverage of at least 95% uptake of two doses of measles-containing vaccine is deemed needed to induce herd immunity to measles [6]; this has been achieved in many countries and as of 2020, 81 countries were declared measles free [7].

However, importation of cases and pockets of under-vaccinated groups remain important sources of infection hindering elimination status and causing measles to re-emerge in countries where it had previously been eliminated [2, 8, 9]. Lo and Hotez [10] pointed out that a 5% drop in vaccine coverage will increase measles cases threefold. Strategies to increase measles vaccine uptake have most often targeted the younger population and their caregivers, anti-vaccination communities, and the vaccine-hesitant group, overlooking the role of healthcare workers (HCWs) we seek care from as an important source of transmission.

Nosocomial measles transmission—measles acquired in healthcare settings—is an important mode of the measles outbreak. Up to 50% of measles outbreaks have reportedly occurred in healthcare facilities, with an increasing trend in recent years [2, 11]; however, the contributing factors for this trend are still not fully understood [12]. HCWs are the frontliners in healthcare delivery and are noted to be 2 to 19 times at higher risk of contracting measles than the general population [13–15]; consequently, infected HCWs can act as vectors for measles transmission to susceptible colleagues and patients who are at an increased risk of developing severe complications due to age, underlying medical conditions and immunocompromised state. Despite the World Health Organisation's (WHO) advocated measures in the 1990s to reduce nosocomial transmission, which encompassed maintaining robust measles immunisation within communities, ensuring sufficient measles immunisation for hospitalised patients, promptly isolating suspected measles cases upon arrival, and reporting to health authorities, outbreaks have continued to occur, irrespective of a country's status in measles elimination [12]. For example, in the USA, Steingart et al. [14] reported a patient infected by an unvaccinated HCW who required intensive care for four days. Another study described how all 14 children who acquired measles in a South African hospital had complications; two children died [16]. Nosocomial transmission must be highlighted because it disrupts care delivery, is costly, and poses a significant health burden with higher associated mortality and morbidity [2, 17]. This paper aims to identify the contributory factors for outbreaks in healthcare settings; and review the control measures that have been implemented, focusing on HCWs. This information can guide policymakers—at the hospital and national level—to implement and evaluate policies to curtail nosocomial transmission.

## Materials and methods

We systematically searched the literature using the population, intervention, comparison, and outcome (PICO) strategy to identify keywords. The search terms included measles, morbilli, rubeola, outbreak, nosocomial, healthcare workers, healthcare personnel, hospital, and healthcare settings. We searched PubMed, Embase via Ovid, Google Scholar, and CINAHL on the 17th of May 2023 using the search strategy developed. We did not set any time limit; only full-text articles in English are included. After eliminating duplicates, two reviewers (AL and IT) independently assessed the search results initially by titles and abstract, followed by a full article review to identify eligible studies. Any disagreements were resolved by consensus with the third author (HB). The reference list of eligible publications retrieved was hand searched to identify potentially relevant studies. In addition, we also searched governmental and international health agencies (e.g. UK Health Security Agency (UKHSA), WHO, and CDC).

## Results

**Fig. 1:**
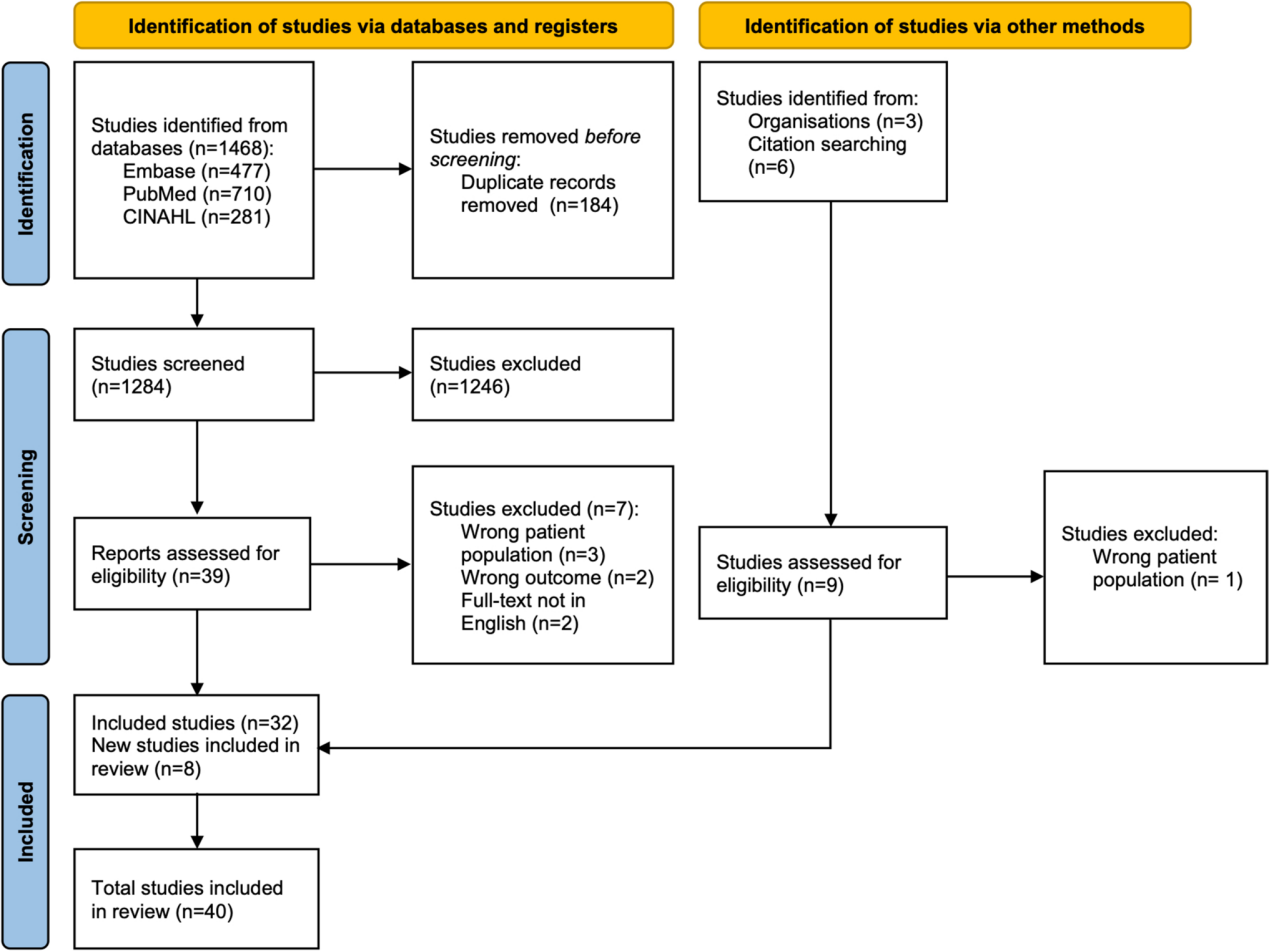
PRISMA flow diagram of the study selection process

Our search retrieved 1468 records. After removing duplicates and screening the articles, 32 studies were eligible. Another 8 additional publications were found by cross-referencing the full-text articles, and 40 articles corresponding to our subject were included in this review (Fig. 1).

### Contributory factors for measles outbreaks in healthcare settings

#### High transmissibility

Measles has the highest basic reproduction rate (*R*_0_) among all vaccine-preventable diseases, typically ranging from 12 to 18 [1]. This indicates that any measles-infected person could potentially infect 12–18 susceptible individuals within a population. The review identified a wide range of reported R_0_ values from 1.4 to 770. This variation is influenced by various factors, including the sociodemographic of the study population (including population density, immunity levels, cultural practices, and disease control measures) as well as the specific strains and temporal patterns of the measles virus [1, 18]. It should be acknowledged that there are currently no standardised methods for estimating R_0_ [1], which may account for the observed variation in values. Accurate estimation of measles *R*_0_ is crucial as it determines the threshold required to achieve herd immunity and, subsequently, the necessary vaccination coverage; therefore, obtaining setting-specific *R*_0_ estimate calls for high-quality surveillance and census data [1, 19].

#### High intensity exposure

The measles virus spreads rapidly in enclosed settings, surpassing tuberculosis and influenza in its transmission rate [20]. This is concerning for HCWs who work in higher-risk areas such as emergency rooms, internal medicine, maternity and paediatric wards, and waiting areas [11, 21–24]. These environments are often crowded, leading to an increased risk of acquisition of infection. The measles virus can remain contagious in the air or on surfaces for up to two hours after an infected person has left the area [25], emphasising the potential for transmission without direct contact with cases and the need for proper ventilation throughout healthcare facilities. The emergency department has been extensively documented as a hotspot for measles transmission due to the proximity and extended periods that patients and visitors congregate in the area [2, 26]. HCWs working in this setting are at the forefront of providing care, and patients often present during the pre-rash stage when the infectivity is highest, yet the disease might not be suspected [11, 13, 27, 28].

#### Delayed care

Untimely diagnosis of measles resulted from several factors: first, as previously mentioned, patients often exhibit non-specific symptoms prior to the onset of rash where index of suspicion for measles is low. This is complicated by atypical disease presentations, particularly in individuals who have previously received any dose of measles-containing vaccine [29, 30] and in immunocompromised patients where the absence of rash has been reported [31, 32]. Measles cases in adults have been reported to elicit more subtle symptoms and are often missed [33]. The lack of recognition or awareness of measles cases in countries with measles-free or near-elimination status, especially among younger HCWs [2, 27, 29, 33], contributes to the problem.

Second, the choice of diagnostics plays a role. The immunoglobulin M (IgM) assay is the reference standard as recommended by the WHO [34, 35]. However, caution is needed as false-negative results may occur when samples are collected before IgM becomes detectable, and false positives can arise due to interference from factors such as rheumatoid factor or other underlying conditions. Additionally, the challenge of false-positive results has emerged as a significant concern in countries that have attained measles elimination, particularly when facing lower positive predictive values [36–38]. On another note, vaccinated individuals in Lebanon [39], China [40], and the United States [27] who have contracted the disease exhibited low or absent IgM responses, further complicating the diagnosis in the post-elimination era [28]. As such, to bolster case confirmation accuracy, utilising real-time polymerase chain reaction (RT-PCR) for detecting virus RNA, in conjunction with IgM assay, can yield more dependable results, particularly in post-elimination settings. This approach also enables the identification of specific genotypes, serving the purpose of enhanced surveillance, although it may be more costly and not readily available in many settings [27, 35, 37, 39, 41]. Limited availability of testing kits could also hinder the confirmation of diagnosis, as in Lebanon [39]; thus, routine stock checks should be performed. Third, a shortage of personnel in all aspects of care, including laboratory and contact tracing teams, compounded the problem [39] and led to delays in instituting the infection control procedures.

#### Failure to use protective equipment and implement control measures

Non-recognition of measles cases prompted ongoing transmission, as evidenced in Arizona, where measles was imported via a hospitalised traveller, eventually leading to a 14-case outbreak. Surprisingly, only one of the 11 measles patients who had accessed care was masked and placed in isolation despite all being infectious [27]. As discussed previously, the highly contagious nature of the virus and its ability to linger in the air for extended periods pose risks to HCWs and hospital visitors. Unknowingly, HCWs continued to have direct, face-to-face interactions with undetected cases without using personal protective equipment (PPE). Consequently, HCWs must adhere to standard precautions while delivering medical care consistently.

#### Vaccine failure

Vaccine failure can arise from factors related to the vaccine itself (operational factors) and host-related factors. Operational factors, encompassing issues like disruptions in the cold chain system, insufficient vaccine doses, inadequate interval between doses, or inadequate vaccine handling such as incorrect reconstitution or the use of expired vaccine, hold greater global significance [42]. However, host-related factors also contribute to an unknown proportion of vaccine failure. Studies indicate that as many as 10% of healthy individuals may fail to respond adequately to routine vaccines, as evidenced by the absence of seroconversion [43]. Other studies have documented cases of measles infection occurring in individuals who were previously vaccinated (secondary vaccine failure) presenting with symptoms similar to those in naïve individuals but milder [28, 41, 44–46], likely attributed to waning vaccine-derived immunity. Interestingly, measles cases were found to be more common in the younger, vaccinated population [11, 21, 44, 47], exemplified in the work of Liu and colleagues [22] that highlighted lower seropositivity for measles antibody in the youngest group. The most posited explanation for this was the reduced likelihood of immune boosting from natural infection in younger adults in measles post-elimination settings [44–46]. Nonetheless, twice-vaccinated individuals experienced faster viral clearance, were less contagious, and exhibited reduced clinical severity compared to unvaccinated cases [41, 46].

#### Unclear immunisation history and lack of registries

A recurring theme in numerous studies is the increased susceptibility of HCWs to measles due to uncertainties surrounding their immune status. This can be attributed to the absence of vaccine registries in hospitals or the loss of vaccination cards [26, 46]. Even when registries were available, there were inconsistencies in data documentation and storage, with some records being stored electronically, while others were managed manually [26, 29, 48]. In some instances, vaccination histories were obtained through self-reporting, which tends to overestimate vaccination coverage [45]. Consequently, during outbreaks, some healthcare centres faced challenges in retrieving accurate vaccination records, causing delays and disruptions [48, 49]; other centres performed emergency serological testing and vaccination of serosusceptible individuals [27, 46, 50]. Notably, many HCWs were either aware of being unvaccinated or were uncertain about their vaccination status [26, 45, 49–52]. For example, only 2.9% of HCWs in a Chinese hospital had received a two-dose measles vaccine, while 41.2% were aware of being unimmunised [45]. Similarly, a Spanish study found that 66.3% of HCWs were not vaccinated [26]. In the case report of a measles outbreak in the United Kingdom, none of the 31 HCWs who were in contact with an index case (a general practitioner) knew their immune status during exposure [49]. These findings highlight the presence of susceptible pockets within the population and underscore potential challenges in accurately determining national measles vaccine coverage and achieving measles elimination.

#### Lack of uniform recommendation on HCW measles vaccination

WHO has recommended that all HCWs who come into contact with patients should be protected against measles, and documentation of immunity—either written proof of receipt of 2 doses of measles-containing vaccine (MCV) and at least one dose of rubella-containing vaccine or a positive immunoglobulin G (IgG) test result—should be required for employment [53]. The Advisory Committee on Immunization Practices (ACIP) made a similar recommendation in 1987, but such recommendations have not been consistently implemented in all healthcare settings [29]. Among the 36 European countries, eight did not have measles vaccination policies in place by 2018, and only five mandated measles vaccination for HCWs [54]. The remaining European countries, as well as other countries including China, Korea, Taiwan, and the United States [14, 21, 22, 40, 54], only recommend HCWs to be vaccinated against measles. However, it is essential to note that documentation of measles immunity should not be considered absolute, as the antibody response can vary among different measles variants and individuals [29, 39]. This was evident in the Korean outbreak, where a population with high vaccination coverage still experienced cases [21], suggesting that relying solely on vaccination history or serologic titres may not be precise enough to determine immunity [29, 38].

Another approach commonly used to establish an individual’s immunity to measles is by considering their birth year. Various studies have reported a wide variation in the birth year used as a threshold to determine immunity, though this is based on the national epidemiological data. For example, the United States considers individuals born before 1957 as immune [14], while Germany [48], Spain [26], and the Netherlands [46] used the years 1970, 1971, and 1976, respectively. Although individuals in these birth cohorts are deemed immune, some studies have shown this was not always the case [14]. Moreover, these country-specific thresholds for immunity pose challenges for countries without similar data, highlighting the need for a broader scope of serological screening to identify serosusceptible individuals.

### Strategies employed to tackle measles outbreaks in healthcare settings

#### Early notification of outbreak and contact tracing

When a measles outbreak occurs, the initial step should be to promptly inform both the hospital’s infection control unit and the local health authorities, as measles is a notifiable disease in nearly all countries [21, 26, 39, 55]. For example, email notifications were sent to all hospital employees once the outbreak was confirmed in Lebanon [39]. In Portugal, daily situation reports were shared with HCWs in the hospital to identify potential contacts, while nationally, health authorities alerted all hospital networks to implement active surveillance [44]. These actions prompted the response team to investigate the source of the outbreak and identify potential contacts [56] while offering prophylactic measles vaccine to unvaccinated contacts [23, 26] and also installing isolation precautions as a preventive measure.

#### Triaging all cases and setting up dedicated isolation unit

Immediate triaging for any incoming patients displaying symptoms of measles is beneficial for preventing widespread nosocomial exposure but also facilitating targeted screening and case detection [23, 29, 39]. Referrals from the community or other hospitals can be pre-screened over the phone to minimise interactions [21, 45, 57]. Any suspected measles case can be placed in isolation, ideally within a negative pressure ventilation room where available, while awaiting laboratory results [21, 27, 39, 44, 58]. These screening units and isolation wards should be situated in a designated area separated from the rest of the hospital to avoid extensive decontamination measures, and proper ventilation is crucial [3, 55]. In Italy, to alleviate the burden on the hospital, only individuals displaying signs of measles complications were admitted for inpatient care [58].

#### Strengthening PPE and physical measures

Another critical measure for controlling the spread of measles is to enhance the use of PPE among all HCWs, especially those directly involved in patient care. This includes making the use of appropriately sized N95 masks mandatory, which has the potential to prevent 100% of all HCW-associated exposures regardless of their immunisation status [21, 27, 29, 39]. Eye protection, gloves, and gowns are also essential components of PPE [2]. Additionally, promoting hand hygiene practices, such as using alcohol-based gel and maintaining physical distancing, are recommended to mitigate measles transmission [17, 44].

#### Improving case detection

During outbreaks, conducting a more sensitive investigation that includes all patients presenting with fever and rash is recommended [26, 50, 58]. A high level of suspicion should be raised in individuals with a recent history of international travel or contact with measles cases. The history of prior measles vaccination and overall health status should also be noted to assess the disease severity. In Korea, throat swabs were collected after exposure to effectively limiting secondary transmission [21]. However, the limited availability of testing kits contributed to delays in identifying cases in Lebanon [39]. On this ground, healthcare facilities should routinely check the availability of diagnostics kits and ensure an adequate supply of vaccines and immunoglobulin, especially when a case is detected [22, 44, 55]. To expedite test results, the Taiwanese hospital has also increased the frequency of laboratory testing, enabling timely diagnosis within a few hours [22].

To ensure HCWs are working optimally, daily health surveillance can be performed during outbreaks, and any potential case with compatible symptoms should be promptly identified and isolated pre-emptively until confirmed negative by RT-PCR [17, 21]. Enhancing the measles surveillance system and timely reporting to health authorities are pivotal steps for successful elimination programmes [17, 45]. Continuous and periodic education and training, such as those provided by CDC, can be utilised to educate HCWs and align the measles case definition for early recognition and timely management of cases especially in a post-elimination era [17, 29, 45, 55]. This approach has successfully controlled past outbreaks in the United States [55]. The US Department of Health issued a notice of possible measles cases before the outbreak, prompted education for HCWs, and employed additional screening questions. Any case detected in one healthcare setting should be disseminated to other healthcare networks to alert fellow HCWs [26, 30]. Awareness campaigns should target HCWs, patients, and caregivers about measles symptoms, the importance of vaccination, and isolation measures.

#### Determining immunity status of HCWs

As previously mentioned, HCWs are a potential source of measles transmission, often unaware of their immune status. As such, proof of two-dose vaccination is recommended as a prerequisite for employment, discouraging reliance on recall alone as sufficient evidence [40]. Incoming and current HCWs, including permanent staff and support personnel like cleaners, security, students, and volunteers, should undergo serologic testing for measles immunity; coordination with relevant authorities is essential for this comprehensive approach [14, 23, 26, 30]. This aligns with UKHSA recommendations, which advise the documentation of either a two-dose MMR or positive serology testing as satisfactory evidence of immunity [59].

Similarly, the Strategic Advisory Group of Experts (SAGE) on immunisation advocates that all HCWs should demonstrate immunity to measles through verification of immunity and/or vaccination [60]. Proof of immunity is mandatory for HCWs in direct patient contact before entering contractual agreements or participating in training programmes. Individuals lacking proof of a two-dose measles vaccine, and those with seronegative or equivocal measles IgG titres, should be offered vaccination [21, 48, 61], as it stands as the most effective preventive measure against nosocomial measles transmission [21, 45]. In resource-constrained settings, age can serve as a proxy to assess measles immunity, utilising a birth year cut-off with country-specific thresholds for maximum effectiveness.

SAGE recommended that countries integrate their surveillance, demographic, and seroprevalence data with vaccination coverage information, tailoring the analysis to local standards to understand the age distribution of susceptibility [60]. For individuals who have never received any dose or are unsure, two doses of MCV-vaccine are administered, while those previously receiving only one dose receive an additional dose. Although HCWs with documented history of two doses of MCV require no further action, assessing IgG titres could be beneficial to confirm immunity and exclude potential vaccine failure, with the option of providing one additional dose for a boost. Post-vaccination, reassessing IgG titres is recommended [22, 48], acknowledging that antibody concentrations may not always align with the level of protection due to the unaccounted cellular immunity [62]. While implementing these measures may be costly, time consuming, and labour intensive, studies indicate their cost-effectiveness compared to controlling a measles outbreak without such interventions

[13, 27].

#### Establishing policy for measles vaccination for HCWs

Policies encouraging HCWs to be vaccinated against measles is an important strategy towards achieving measles elimination, especially in countries that are still without one, such as in Lebanon [39]. Adoption of a more stringent vaccination policy could ensure all HCWs have the optimal level of immunity, bearing in mind there must be a pre-defined criterion for evidence of immunity as not all HCWs require vaccination (i.e. if acquired prior natural infection) [13]. Also, it should be noted that evidence of measles immunity significantly lowers but does not eliminate the risk of acquiring the disease [29, 41]. Thus, evidence of immunity is not absolute, and the potential risk of measles infection persists due to the possibility of vaccine failure [22, 38]. Liu et al. [22] proposed serologic screening of HCWs prior to vaccination and offering vaccines at cost price for individuals with seronegative results. Implementation of their pilot strategy for two months indicated 99.4% of all hospital employees were seropositive or had been revaccinated, noting the strategy to be very effective and cost efficient. Breakthrough cases still happen despite sufficient vaccination in a population; however, Song and colleagues [21] hypothesised that high vaccine coverage would reduce the size of the outbreaks. Although there is a clear need for a strengthened policy for HCWs, vaccination practices are not solely determined by policies, as other factors can influence vaccination rates, such as education and political support [2], personal knowledge and attitudes towards measles vaccines, and communication between leadership and HCWs [63]. Furthermore, measles vaccine policy for HCWs does not guarantee implementation [13]. Priority should be to enforce vaccinations of HCWs in high-risk settings (emergency rooms, infectious diseases, maternity and gynaecology, paediatrics, and cancer units) [22], and any HCWs that refuse to get vaccinated can be reallocated to a lower-risk setting [17]. Strategies to improve vaccine uptake include ward-based vaccination clinics at convenient times and employing visible immunisation champions [49].

Measures to increase vaccine coverage, such as free measles antibody testing, increasing testing speed, and free vaccines for susceptible HCWs, have been suggested [22]. Another solution proposed was to hold a one-off catch-up measles vaccination programme, similar to that for influenza [13, 51], employing the bottom-up and top-down approaches for its successful implementation [51]. Monitoring vaccine coverage data is vital to ensure measles immunity in HCWs [13], and proper documentation is paramount. Occupational health teams should maintain a complete and updated record of vaccination status of all HCWs, preferably in an electronic registry, to ease accessibility and retrieval in emergency settings [27, 48, 51, 55]. Inadequate records can lead to duplicate testing or over-treatment from additional vaccination, adding unnecessary financial and human resource burden, but also act as a barrier to rapid response during an outbreak [55]. Consequences for non-compliance should be considered, with some suggesting penalties for HCWs refusing vaccination [64]. Nevertheless, hospital managers are crucial to making the safest decisions for the patients, the public, and other HCWs.

#### Management of exposed healthcare personnel

Any unvaccinated HCWs who come into contact with measles-infected patients should be offered post-exposure prophylaxis, including vaccination within 72 hours of exposure or immunoglobulin within six days if vaccination is contraindicated [3, 55]. This practice has been implemented in countries including Germany [48], Spain [30], the United States [15], and Portugal [44], but not in Lebanon [39]. Timely vaccination might help reduce the severity of the disease and minimise the risk of transmitting it to others [27, 30, 41]. Interestingly, all 608 HCWs in China received measles vaccination as an outbreak response, regardless of their vaccination history, and effectively controlled the outbreak [45]. Another more feasible approach was the administration of vaccines only to HCWs without documented prior vaccination [40]. This has also successfully contained the spread of the disease, affecting only 11 HCWs and with no impact on inpatients despite not being targeted by the measles vaccine, suggesting the indirect protection of patients in healthcare settings where a high proportion of HCWs are vaccinated [40]. Furloughing HCWs for a minimum of 18 to 21 days since their last exposure [26, 30, 49–51] followed by strict home isolation [39] is ideal to prevent 91% of measles exposure, as highlighted by Gohil and colleagues [29]. However, it should be emphasised that furloughing such an extended period in limited staffing settings could add further strain on the hospital operation and costs. Overall, significant efforts are necessary to ensure hospital safety and the reliability of HCWs.

#### Developing a pre-incident response plan

The United States successfully controlled a measles outbreak by having a pre-incident plan and conducting regular simulations, as documented by Fifolt et al. [55]. This plan encompasses patient flow management, notification guidelines, environmental decontamination protocols, and timely post-exposure prophylaxis and isolation guidance. Reviewing and updating these plans regularly to incorporate current guidelines is crucial. Regular drills and simulations are essential to ensure a well-coordinated and effective response during an outbreak, even if only a two-hour table top activity yearly. Additionally, the preparedness efforts should involve a multidisciplinary response team that includes representatives from various sectors, ensuring clear roles and responsibilities for all involved parties.

## Discussion

**Fig. 2:**
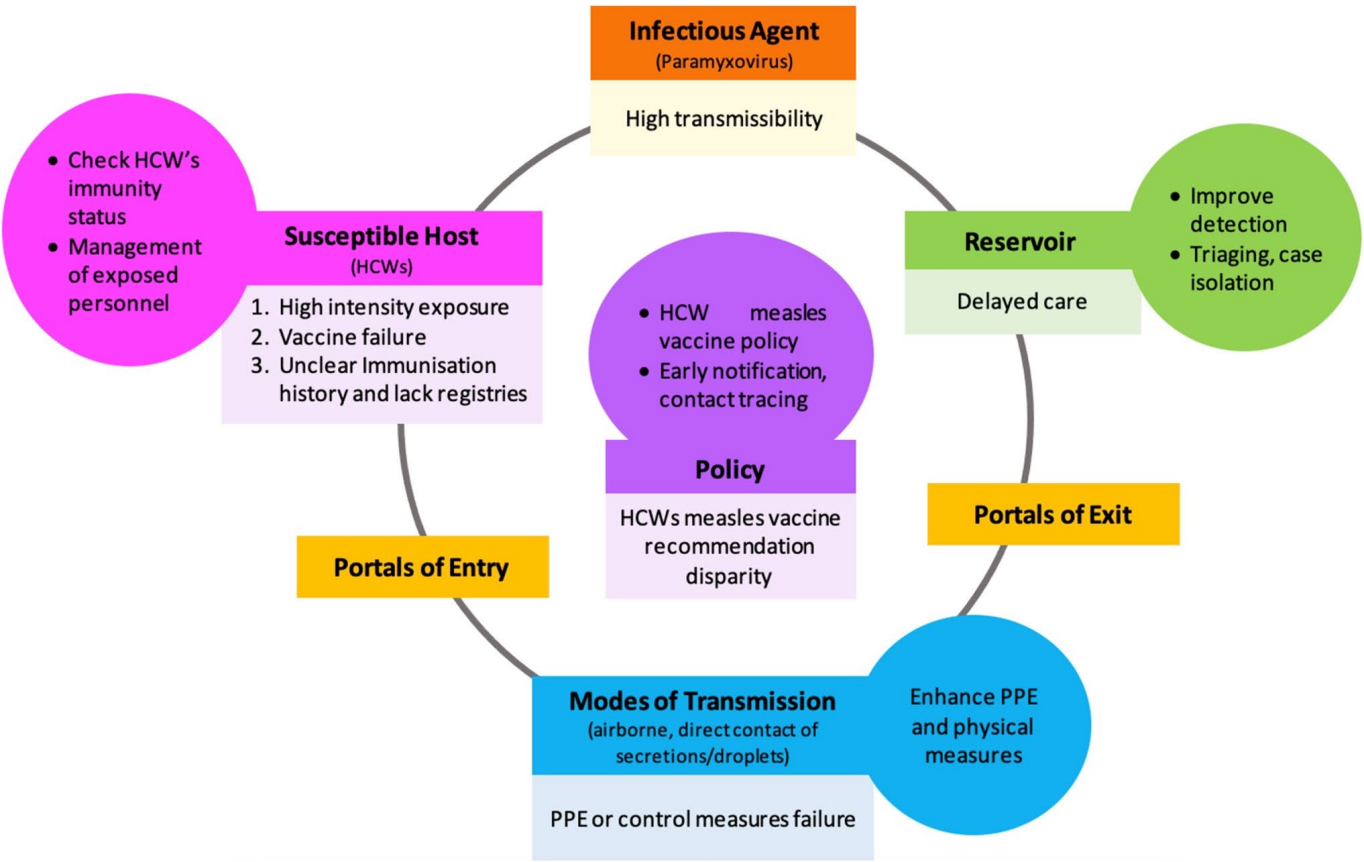
Strategies to minimise nosocomial measles transmission

Although measles vaccination is widely incorporated into national programmes in nearly all countries, there are still identifiable pockets of susceptible individuals within the population, including healthcare workers. Insufficient vaccination coverage among HCWs significantly contributes to the transmission of the disease [26, 45], along with delayed recognition and management of measles cases, vaccine failures, and the inherent nature of the disease. We attempted to identify the factors contributing to nosocomial measles transmission and the strategies that have been implemented (Fig. 2), to provide insights for policymakers and hospital managerial to adopt and adapt to their local settings.

Workplace vaccination against measles for HCWs represents a critical strategy for achieving herd immunity, protecting their unvaccinated colleagues and immunocompromised patients from the disease and its severe complications [17]. Vaccination policies should encompass permanent staff and temporary or contract personnel in healthcare settings, such as travel nurses, the cleaning team, security officers, volunteers, and students. Studies have consistently shown that healthcare students in hospital settings are among the least immunised group [11, 65], possibly due to discrepancies in occupational vaccination recommendations [65]. To ensure HCWs’ immunity against measles, healthcare facilities can conduct serology testing for all HCWs during outbreaks and selectively vaccinate those found to be susceptible. A study in Taiwan employed this strategy and achieved a cost reduction of 69.4% with higher vaccination rate and policy compliance [22]. Implementing this approach in 706 HCWs costs approximately US$5,675, and none of the HCWs were infected during the subsequent outbreak [22]. Although this intervention may be perceived as costly, previous research has reported outbreak control costs as high as US$800,000 in Arizona, involving only seven measles-infected HCWs [27]; half the costs were attributed to furloughing HCWs due to presumptive exposure or lack of evidence of immunity. Measles outbreaks in healthcare facilities entail substantial economic, societal, and healthcare system burdens due to disruption. Therefore, implementing the new strategy observed in Taiwan represents a promising investment to mitigate the potential burden and accelerate measles elimination.

Considerations of the perceptions and attitudes of HCWs towards the measles vaccine are crucial when devising vaccination policies, as studies indicate that their knowledge and confidence in the vaccine’s safety and effectiveness are often lacking [63, 65]. Borggreve and Timen [63] conducted interviews with HCWs responsible for implementing measles guidelines in the Netherlands. They identified barriers like poor inter-departmental communication, social factors like religious beliefs, unclear guidelines, misconceptions influenced by pharmaceutical companies, and fear of side effects hindering implementation. Surprisingly, a survey in a Chinese hospital revealed low confidence in the measles vaccine, with only 31% of 646 staff considering it safe, 36.1% believing it effective, and only 34.5% expressing willingness to be vaccinated [45]. In contrast, a study in France found a high acceptance rate (78.6% willingness) among HCWs [65]. Variations in HCWs’ knowledge may contribute, with some perceiving themselves at lower risk [66] or were ignorant about the risks [22].

In European nations, policies on measles vaccination for HCWs vary, with recommendations in 17 countries, mandatory requirements in 5 countries, and an absence of such policies in 8 [67]. In the remaining countries, like Estonia, Norway, Poland, Czech Republic, and Slovakia, measles vaccination is advised only for specific contexts, such as HCWs in haematology and paediatrics ward in Estonia. This policy diversity among neighbouring countries could potentially impact social cohesion and lead to HCWs migration based on individual preferences [68]. Despite existing recommendations, a review conducted by Maltezou and colleagues [54] noted that only 8.8–62.7% of HCWs had received two doses of measles vaccines, raising concerns about the effectiveness of vaccine mandates in encouraging compliance among HCWs.

The proposition of implementing vaccine mandates on HCWs has sparked ongoing discussion, yet a consensus remains elusive. Despite concerns about potential infringements on individual rights and scepticism regarding motivations, the primary objective of vaccine mandates is to safeguard HCWs, their patients, and the broader healthcare environment. These mandates align with the principles outlined in the Good Medical Practice code and may be deemed an obligation by professional bodies [69]. Furthermore, proponents argue that vaccination policies for HCWs serve to set an example, positioning them as leaders in adhering to sound preventive medicine practices. A successful case of implementation can be observed in a Seattle hospital that introduced mandatory influenza vaccination for all staff, as documented by Talbot et al. [70]. Although met with initial resistance, over four flu seasons, vaccination rates for over 5000 employees increased to more than 98%. Hospitals with mandatory influenza vaccination protocols for HCWs achieved vaccination rates close to 100%, whereas recommending vaccination without a requirement or on-site availability resulted in less than half of HCWs getting vaccinated [71]. To date, no study has examined the impact of measles vaccine mandates on HCWs. While vaccine mandates have proven effective, maintaining an ethical balance is crucial to prevent excessive coercion that may lead to disruptions in healthcare due to shortages resulting from termination due to non-vaccination. Nowalk et al. [72] found that hospitals with mandates and consequences for non-compliant HCWs had nearly double the vaccination rates compared to those without consequences. Peterson and colleagues [73] highlighted effective interventions for increasing vaccine uptake, including providing non-monetary incentives, sending reminders or nudges, hosting discussions with senior leaders, and setting institutional deadlines. For non-compliant personnel, alternative approaches such as active opt-out, exemptions based on medical, religious, or other beliefs, and relocation to lower-risk areas could be considered [13]. This approach aims to address non-compliance while offering individuals the opportunity to continue contributing to healthcare in roles that pose lower risks. Striking a balance between coercion and providing supportive measures is essential for successful vaccine mandate implementation in healthcare settings.

Monitoring measles transmission is of utmost importance and complements the surveillance of vaccine coverage at both country and hospital-specific levels [13]. Although the effectiveness of genotyping has diminished due to the decreasing diversity of the measles virus [74], it remains a valuable method. By 2021, only two genotypes (B3 and D8) were active. The standard protocol involves sequencing the N-450 regions, with current recommendations emphasising the sequencing of as many transmission chains as possible. Additionally, the use of advanced sequencing tools, such as whole genome sequencing, is encouraged. Williams and colleagues [74] highlighted the significance of integrating both molecular and epidemiological data, considering factors like the time between cases and outbreaks, geographical distribution, and phylogenetic analysis, for a comprehensive understanding of measles transmission.

Implementing measles immunisation is a cost-effective public health intervention, but formulating and implementing policies is demanding and resource intensive. In parallel, healthcare facilities need to strengthen their existing systems for reporting measles cases, enhance physical measures to control measles transmission, and establish pre-incident response plans for potential outbreaks. These measures include setting up specialised units to screen patients displaying symptoms such as fever or rash, facilitating the detection of cases in their prodromal stage, and isolating individuals suspected of having measles until proven otherwise [33]. Promoting the use of PPE and emphasising adherence to proper hand hygiene practices among HCWs should be prioritised.

## Conclusion

This review highlights the control measures implemented to address measles infections in response to the challenges that were recognised to underlie outbreaks occurring globally. Despite widespread measles vaccination efforts, susceptible individuals, including HCWs, persist in populations, resulting in nosocomial transmission. A coordinated and comprehensive approach is crucial to promptly identify measles cases, interrupt disease transmission, maintain a high level of immunity against measles, implement effective public health responses, and strengthen infection control measures. By adopting such an approach, HCWs are directly safeguarded, patients are indirectly protected, and the spread of outbreaks can be minimised.

## Abbreviations

ACIP: Advisory Committee on Immunization Practices
HCWs: Healthcare workers
IgG: Immunoglobulin G
IgM: Immunoglobulin M
MCV: Measles-containing vaccine
MMR: Measles, mumps, and rubella
PPE: Personal protective equipment
RT-PCR: Real-time polymerase chain reaction
SAGE: Strategic Advisory Group of Experts
UKHSA: UK Health Security Agency
WHO: World Health Organisation
